# Insights into Antimicrobial and Anti-Inflammatory Applications of Plant Bioactive Compounds

**DOI:** 10.3390/microorganisms11051156

**Published:** 2023-04-28

**Authors:** Gregoria Mitropoulou, Elisavet Stavropoulou, Natalia Vaou, Zacharias Tsakris, Chrysa Voidarou, Arsenis Tsiotsias, Christina Tsigalou, Birce Mercanoglou Taban, Yiannis Kourkoutas, Eugenia Bezirtzoglou

**Affiliations:** 1 Laboratory of Applied Microbiology and Biotechnology, Department of Molecular Biology & Genetics, Democritus University of Thrace, 68100 Alexandroupolis, Greece; gmitropo@mbg.duth.gr (G.M.); ikourkou@mbg.duth.gr (Y.K.); 2 Department of Infectious Diseases, Centre Hospitalier Universitaire Vaudois (CHUV), 1101 Lausanne, Switzerland; 3 Laboratory of Hygiene and Environmental Protection, Department of Medicine, Democritus University of Thrace, 68100 Alexandroupolis, Greece; nvaou@hotmail.com (N.V.);; 4 Laboratory of Microbiology, Department of Medicine, National and Kapodistrian University of Athens, 11527 Athens, Greece; ztsakris@gmail.com; 5 Department of Agriculture, University of Ioannina, 47132 Arta, Greece; xvoidarou@uoi.gr; 6 Department of Obstetrics, University of Western Macedonia, 50200 Ptolemaida, Greece; arsenis74@hotmail.com; 7 Laboratory of Microbiology, Department of Medicine, Democritus University of Thrace, 68100 Alexandroupolis, Greece; 8 Dairy Technology Department, Faculty of Agriculture, Veterinary and Agriculture Campus, Ankara University, Diskapi, Ankara 06110, Turkey; birce.taban@ankara.edu.tr

**Keywords:** antimicrobial activity, bioactive compounds, plant derivatives, human microbiota, antibiotics, antioxidants, inflammatory diseases, medicinal plants, new antimicrobials

## Abstract

Plants have long been thought to contribute to health promotion due to their fiber and phenolic content, as well as their inherent biological potential. The bioactive derivatives of medicinal plants are a valuable resource in the fight against serious diseases all around the world. The present review focuses on the current state of knowledge on the usage and medicinal applications of plant bioactives. Issues concerning the effect of aromatic plant derivatives on human gut microbiota and their antimicrobial and anti-inflammatory potentials are discussed and worth further exploring.

## 1. Introduction

Plant-derived compounds refer to materials or ingredients that originate from plants. They are secondary metabolites that are involved in the response to exogenous stressors. In the years 75–45 BC, Pedanius Dioscorides (who lived in the first century A.D.) wrote an encyclopedia called *De Materia Medica*, in which he described 600 therapeutic medicinal plants in the form of a series of scientific research [1]. Since then, a high number of herbal drugs have been recognized as potential therapeutic agents. Conventional medicine, the so-called Western medicine, retains the lion’s share, but a growing interest in herbal treatments is becoming more obvious. It seems that herbal treatments can play an important role in improving human health, which has led to the growing interest in alternative therapies and the therapeutic use of plants.

Low- and middle-income countries in Africa and America use traditional medicine to help cover their primary health care needs. In industrialized countries, the adaptation of traditional medicines is termed “alternative” medicine. The global market for herbal medicines currently stands at over USD 80 billion annually and is growing steadily. A significant asset is their low cost in comparison to synthetic industrial forms of medication. Until now, synthetic drugs were widely accepted due to their fast-acting effects. However, doctors and patients have begun to realize the benefits associated with natural remedies.

Despite the intense interest in the field, the mode of action is still under investigation as there is a plethora of drastic chemical substances isolated from plants such as alkaloids, phenols, tannins, glycocides, and terpenoids. The role of medicinal plants in human health is clearly enormous. The World Health Organization (WHO) lists 252 medications as fundamental and essential, 11% of which are completely of plant origin, and a considerable proportion of which are synthetic drugs derived from natural precursors [2]. Namely, Digoxin was obtained from *Digitalis* spp., quinine and quinidine were collected from *Cinchona* spp., vincristine and vinblastine were obtained from *Catharanthus roseus*, atropine was obtained from *Atropa belladonna*, and morphine and codeine were obtained from *Papaver omniferum*. In addition, natural origin medications account for 60% of anti-tumor and anti-infectious pharmaceuticals that are now on the market or in clinical trials [3].

Medicinal plants provide chemicals for the creation of new medications, biomimetic synthesis, and the discovery of new therapeutic qualities not previously associated with known molecules. In most circumstances, medicinal plants’ crude extracts can be utilized as medicines.

## 2. Gut Microbiome as a Target of Aromatic Plant Bioactive Derivatives

### 2.1. Gut Microbiome in Brief

The term microbiome refers to the collective genomes of microorganisms in a specific environment, whereas microbiota refers to the community of microorganisms themselves. The human gastrointestinal tract contains approximately 100 trillion microorganisms (mostly bacteria, but also viruses, fungi, and protozoa)—the microbiome is now best thought of as a virtual organ of the body. The human genome contains approximately 23,000 genes, whereas the microbiome encodes over 3 million genes that produce thousands of metabolites that replace many of the host’s functions, influencing the host’s fitness, phenotype, and health [4]. The intestinal microbiome represents a whole world of bacteria, fungi, archaea, viruses, helminthes, and parasites that have co-evolved with humans for thousands of years. Their diversity and abundance retain a balance called eubiosis, which is significant and critical for humans’ well-being. The opposite condition of misbalance, called dysbiosis, caused by factors such as age, stress, antibiotics, diet, etc., could trigger inflammation and propel chronic diseases. Therefore, the maintenance of a functional gut microbiome through numerous interventions, including nutraceuticals, is of paramount importance.

### 2.2. The Most Known Actions of Medicinal Plants on Intestinal Microbiota

As technologies used to study the gut microbiota have improved, the relationship between the gut microbiota and health has become increasingly obvious. Recently, there have been an increasing number of studies that investigated the interaction between the gut microbiota and herbal medicines, which occurs primarily through two pathways. One pathway is that the gut microbiota “digests” the herbal medicines into absorbable active small molecules, which enter the body and induce physiological changes. The other pathway is that herbal medicines regulate the composition of the gut microbiota and its secretions, thereby changing the gut microbiota and its secretions and inducing physiological changes. In summary, the interactions between the gut microbiota and herbal medicines can be attributed to absorbable active small molecules and changes in the gut microbiota and its secretions [5].

### 2.3. The Influence of the Drastic Substances on Immune Response

Research shows that aromatic and herbal plants are bestowed with functional ingredients that may have immunoregulatory potential [6,7] and provide protection against pathogenic invaders. They could be used as an adjunctive treatment for boosting the immune system, enhancing the mucosal barrier function, and inhibiting bacterial adherence and invasion capacity in the intestinal epithelium by being in direct antagonism with pathogens [8].

The pathogenicity of bacterial and viral infections closely related to the gut-lung axis is involved, as the intestinal microbiota boosts alveolar macrophage activity, thus having a protective role in host defense against lung disease [9]. In this vein, it is supported that there is a permanent “dialogue” between the gut, lung, and brain as a whole communication along a complex immunologic inflammatory neuroendocrine and neural network system, the so-called gut-brain-lung axis [10]. Yet, due to their anti-inflammatory potential [11] and the capacity of free radical scavenging, herbs are ascribed to be functional against cancer activation [12]. As known, gut microbiota is associated with immune response [8,13]. The deployment of the innate immune response is dependent on pattern recognition receptors (PRRs) [14]. The plant chemicals seem to manage the innate immune cells via an interplay process between the cell wall components or the metabolites and host PRRs [15]. Still, these components could activate dendritic cells (DCs) and macrophages, boosting adaptive immune responses (B-cell differentiation, T-cell homing, Th17 cell stimulation) [16]. Pattern recognition receptors (PRRs) have a quite crucial role in the innate immune response by identifying microbe-associated molecular patterns (MAMPs) which are specific to each pathogen and essential for its viability. PRRs are proteins that act as exogenous signals associated with pathogen inhibition [16]. They trigger pro-inflammatory signaling pathways, phagocytic responses, or bind to pathogenic invaders [13],. Additionally, the PRRs recognize DAMPs (danger-associated molecular patterns), including Toll-like receptors (TLRs) and NOD-like receptors (NLRs), which are expressed on the surface of sentinel cells, as danger signals that are produced by damaged or necrotic host cells that enhance the pro-inflammatory response [15]. In this line, as PAMPs are recognized by PRRs, due to the activation of sentinel cells (macrophages, epithelial cells, mast cells, and dendritic cells), the expression of chemical messengers such as cytokines and chemokines are produced for the regulation of further innate immune response [14]. Lastly, cytokines should activate the antigen-presenting cells and specific adaptive immunity by eliminating pathogens [15,16].

The role of cytochromes P450 (CYPs), which are enzymes expressed mostly in hepatic and intestinal tissues [17] of the human body, is another important issue. Cytochromes P450 can metabolize an array of xenobiotic substances, aromatic and herbal plants, and medications. Clearly, the colossal metabolic activity of the human gut microbiota is linked to its pool of CYP enzymes, which catalyze phase I and II reactions in drug metabolism. CYP enzymes actions bear a resemblance to those of pro-inflammatory cytokines and IFNs. Moreover, they are involved in the initiation and persistence of pathologic pain by directly activating sensory neurons and inflammatory cytokines [17].

**Figure 1:**
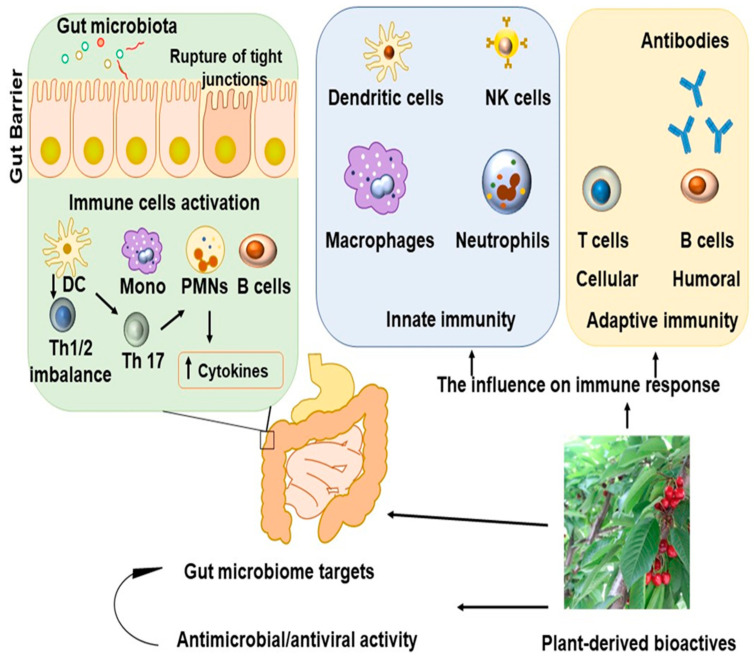
Plant-derived bioactives target gut microbiome, leading to antimicrobial–anti-inflammatory activity through immunomodulation.

Herbal and medicinal plants influence many cytokines. Various infectious disease states are characterized by the expression of soluble extracellular cytokines or glycoproteins in the form of interferons, interleukins (ILs), and chemokines [14,15,16]. Following inflammation, cytokines are secreted via intermolecular dialogues to sustain physiological stability in cells (Figure 1).

## 3. Herbal Plants and Antimicrobial–Antiviral Activity

### 3.1. Mode of Action

The mechanisms of the actions of phytochemicals against bacteria have not yet been completely elucidated, as there is evidence of variability in relation to their molecular basis for their modes of action [17], including cellular membrane rupture, inhibition of bacterial growth, modulation of signal transduction, and gene expression pathways or meddling in microbial metabolic processes. Phytomedicines have shown bacteriostatic or bactericidal activities in different gut pathogens. Their activity was mainly attributed to the presence of flavonoids and alkaloids, while terpenoids, glycosides, tannins, and quinones have promising health effects [17].

**Figure 2:**
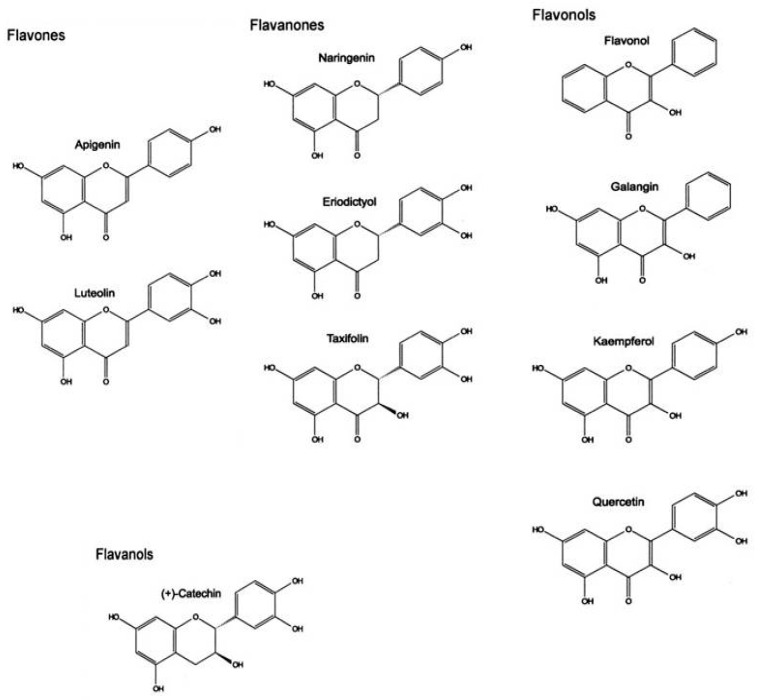
Chemical structure of flavonoids.

Flavonoids (Figure 2) are a group of polyphenols with antioxidant or free radical scavenger activities and anti-mutagenic and anti-carcinogenic properties due to their capacity to regulate cellular enzyme function [18]. Catechins are flavonoids that are found in green plants with inhibitory effects against *Vibrio cholerae*, *Streptococcus mutans*, *Shigella*, and some viruses [19]. Alkaloids mainly act against gut pathogens, but they are also used as psychotropic drugs [18]. Among glycosides, the most known are digitoxin from *Digitalis*, barbaloin from *Aloes*, and salicin from *Salix*, which are all used for digestive purposes.

Tannins are mostly used as chemicals and antiseptics. Tannins possess the sesquiterpene lactones that are shown to have antibacterial, anthelmintic, and antiprotozoal action [20]. Terpenoids are active against bacteria, fungi, viruses, and protozoa. Quinones have a potent antimicrobial effect against *Bacillus anthracis*, *Corynebacterium pseudodiphthericum*, and *Pseudomonas aeruginosa* [21].

### 3.2. Antimicrobial Resistance (AMR)—The “Superbugs”

Amidst their beneficial potential, the emergence and spread of antimicrobial resistance (AMR) in plants are witnessed. As known, bacteria are involved in the transfer of antimicrobial resistance genes (AMGs), which is not limited to foodborne pathogens, but also includes environmental microbes that are involved in various agricultural sites and may be transmitted [22,23]. The microbes that exhibit multi- or pan-drug resistance, the so-called “superbugs”, have become an unprecedented threat for our world and all the conquers of science in recent years.

Nevertheless, there is limited knowledge in the field of agriculture. Certainly, bacterial soil communities, manure, processing procedures, wastes, and irrigation water can carry ARGs that cultured plans will uptake through passive absorption or water transport [24]. Factors such as the plant growth stage, specific plant characteristics and constituents, biotic stresses, climate, as well as antimicrobial agents (AMAs) activity should be taken into consideration [23]. The transfer of antibiotic resistance was observed in raw salads and vegetables [25,26,27,28]. Hopefully, AMAs are not used in plant production to the extent that they are used in animal or human medicine. Streptomycin, which is used to treat apple and pear trees from the phytopathogenic bacterium *Erwinia amylovora*, seems to be strongly sequestered in soil [23]. As is known, the irrational extensive use of antibiotics has led to an increase in the rate of resistant strains of bacteria in different ecosystems [29,30]. Over and above, hospital, industrial, and domestic sewages are reservoirs of antibiotic-resistant bacteria that may intersperse on other recipients in different ways.

As a result, “*pathogenicity islands*” are formed, harboring multiple drug resistance genes [30]. The study of a new antibiotic, tigecycline, clearly showed the importance of the issue of the antibiotic spreading into the environment [31]. The authors compared similar bacterial strains for antibiotic resistance, and a higher resistance was revealed in the environmental ecosystem. Concerning tigecycline, a rapid spread in the environment was found, even during a short period of use in clinical practice [27]. In Switzerland, a global antibiotic surveillance study showed the huge quantities (49.250 kg) of AMAs used in veterinary practice, even after the 1999 ban. As known, AMAs used in animal practice for prophylaxis or therapy must be different from those for human use to limit antibiotic spreading. However, most antibiotics used in veterinary practice have similar or even identical structures to those prescribed in human practice [23]. Systematic monitoring, surveillance, and educational campaigns about the spread of antibiotics must be performed across various ecosystems to decipher the interconnections and routes of transmission, optimize sewage treatment, and safeguard public health.

Various aromatic plants and herbs are composed of diverse chemical constituents. Therefore, each one must have a different underlying mechanism of action on human health. Herbal plants are found to modulate components of an innate or acquired immune response due to their secondary metabolites, which have immunomodulating capacity [6]. The importance of the spread of antibiotics should not be underestimated in order to obtain quality herbal products.

### 3.3. Examples of Plant Derivatives and Their Targets

Nutraceuticals are medicinal foods made from herbal or botanical raw materials that boost health, regulate immunity, and thereby prevent or treat different types of acute or chronic diseases. Due to the increasing resistance of classic antibiotics against pathogens, they monopolize the interest in their use as an alternative or adjuvant therapy in specific disease states. However, the plant growth stage, specific morphological characteristics, geographical area, biotic stresses, and culturing conditions should play an important role in gaining the proper and marketable chemotype [32]. In this line, the important antimicrobial activities of essential oils (EOs) produced from aromatic plants were revealed with a potential interest for prophylaxis or therapy [32]. The effect of EOs produced from aromatic plants on the seizure latency and severity of pentylenetetrazol (PTZ)-induced seizures in mice was shown. Yet, the mice that were administered *Mentha piperita* showed no seizures or survival [33]. Research shows that the main potent component of *Mentha* is essential oil, although high polymorphism and variation in components are observed among the different EOs in *Mentha* [34].

The pharmaceutical industry is seeking phytochemical compounds and extracts as alternative therapeutic antiviral drugs. Research on natural treatments against coronaviruses SARS-CoV and MERS was enrolled. *Lycoris radiata*, *Pyrrosia lingua*, *Artemisia annua*, and *Lindera aggregate* showed promising antiviral effects [35]. *Amaryllidaceae species* contain lycorine, which showed a potent effect on SARS-CoV [35], but also on *Herpes simplex virus* (HSV, type I) [36] and *Poliomyelitis virus* [33]. Yet, the root extract of *Potentilla arguta* blocked respiratory syncytial virus (RSV) [35]. Recently, this research was extrapolated to SARS-CoV-2, as the virus belongs to the same family and presents similarities [35]. Antioxidant and free radical scavenging capacities were attributed to black tea flavonoids neutralizing bovine coronaviruses infections [37]. Furthermore, the studies of the plant extracts have documented their antiviral action [35] by inhibiting in vitro and in vivo viral replication [38,39].

The plant extracts showed potential antibacterial activities against sensitive and resistant pathogens via different mechanisms of action, as summarized in Table 1.

The antibacterial activity is carried out by two main mechanisms. The first mechanism includes a chemical interference with the synthesis or function of vital compounds in the bacteria, such as bacterial protein biosynthesis, bacterial cell wall biosynthesis, bacterial cellular membrane destruction, bacterial DNA replication and repair, and inhibition of a metabolic pathway. The second mechanism focuses on the bypassing of the conventional antibacterial resistance mechanisms, such as efflux pumps, destructing antibiotic enzymes, or enzymes changing the antibiotic structure.

## 4. Anti-Inflammatory Applications of Herbal Compounds

As discussed above, EOs could be a promising alternative therapy to temper oxidative stress, provided that they are free radical scavengers, metal chelators, and mighty antioxidants [53] due to their components. Terpenes, monoterpenes, and sesquiterpenes, as well as allyl- and propenylphenols, are most frequently identified in EOs, although their metabolic pathways remain under investigation [54,55].

Nevertheless, a recent study trying to explain the mechanism of action of EOs showed that they could block the mitogen-activated protein kinase (MAPK) pathways, blocking NF-κB activation by confining oxidative stress, which limits the production of several pro-inflammatory mediators [56].

Due to their potent impact as scavengers of free radicals, there is evidence that EOs successfully prevent brain dysfunction, cancer, heart disease, and immune system disturbances that may result from cellular damage [57]. It is also of note that bacterial phagocytosis occurring during inflammation highly increases the oxygen consumption (superoxide anion radical (O_2_^−^)) that is swiftly converted to hydrogen peroxide (H_2_O_2_) [53,57]. Yet, during the inflammation process, RNS free radicals are generated in the form of nitric oxide (NO) and peroxynitrite anion (ONOO^−^) in activated macrophages and neutrophils. RNS free radicals in high concentrations can cause cellular damages by the nitration of host molecules [58]. The modulation of inflammation status was succeeded by means of medicinal plants as an alternate or adjuvant therapy. Medicinal plants possess anti-inflammatory and immunomodulatory properties, including inhibitory effects on the suppression of cellular and humoral immunity, lymphocyte activation, and propagation of apoptosis. The chemical compounds and activities of medicinal plants, such as curcumin from *Curcuma longa* and epigallocatechin-3-gallate from *Camellia sinensis*, were studied with promising results in the inflammation station of colitis, rheumatoid arthritis, and atherosclerosis [59]. The anti-inflammatory action of curcumin is produced through the Janus kinase JAK-STAT pathway by blocking the synthesis of cytokine IL-12 and relieving the inflammation status, blocking the MAPK pathway, cytokine-mediated NF-κB activation and pro-inflammatory gene expression, the arachidonic acid pathway, and iNOS expression [60]. The JAK-STAT pathway initially uses epigallocatechin-3-gallate from *Camellia sinensis* during the inflammatory process by amending transducers of inflammation, such as the JAK/STAT, NF-κB pathway, but also PI3K/Akt, COX-2/5-LOX, by affecting the AA pathway [58].

### 4.1. Inflammation as the Hidden Flame in a Variety of Diseases

Inflammatory diseases occur from the body’s response following tissue damage. Acute inflammation that fails to be regulated leads to chronic inflammation, predisposing the body to the deployment of cancer, neurodegenerative diseases, or autoimmune diseases [61,62].

The activation of multiple intracellular signaling pathways, cellular receptors, tyrosine kinases, and transcription factors leads to the overexpression of pro-inflammatory genes [62]. In this vein, mast cells and leukocytes attend to the site of the injury and a “respiratory burst” occurs, characterized by intense oxygen uptake and, consequently, increasing the release of reactive oxygen species (ROS) and reactive nitrogen species (RNS) at the site of the injury [63,64] for defusing microbial invaders. Due to the body’s defense antioxidant capacity mechanisms, there is a neutralization of the oxidative stress. However, when ROS accumulate at high levels, the body’s antioxidant capacity is not effective and there is DNA damage as a result of the oxidative stress [65].

### 4.2. EOs as an Alternative Weapon against Inflammatory Pathologies (IBD, TDM2, Obesity)

Traditionally, multiple medicinal plants and herbs such as *Atropa belladona* and *Chelidonium majus* have been used for relieving symptoms of gastrointestinal diseases from dyspepsia to irritable bowel syndrome (IBS) and chronic inflammatory bowel diseases (IBD). Their action includes the enhancement of gastric secretion and the suspension of the spasmolytic, astringent, antidiarrheal, and inflammatory effects [66,67].

As discussed previously, the gut microbiota plays a key role in the development of gastrointestinal diseases. The disturbance of the gut microbiota results in reduced microbiota diversity, leading to the altered production of antimicrobial metabolites and short chain fatty acids (SCFAs). Bacterial overgrowth in the small intestine is found in IBS patients [66].

Chronic inflammatory bowel diseases (IBD), such as Crohn’s disease and ulcerative colitis, occur following gut dysbiosis. Shifting in the microbiota profile with low bacterial diversity and an altered production of SCFAs is observed [68].

When ingested, medicinal herbal plants and their compounds seem to possess a dual role, as from one aspect, they are metabolized by the bacteria of the intestinal microbiota, and from the other aspect, they induce shifts in the microbiota variety and function [68]. However, it is largely discussed and remains to be investigated if this interplay with the intestinal microbiota possesses a causative character or whether these are disease symptoms and disorders [69].

Diabetes mellitus (DM) is an endocrine metabolic disorder with severe complications. Diabetes is caused by either the lack of insulin production by the pancreas, a disease known as type 1 diabetes mellitus (T1DM), or the inability of the cells to react suitably to the insulin produced, a situation known as type 2 diabetes mellitus (T2DM). Over the last years, the incidence of T2DM has increased in developed countries, and beyond the genetic impact, various environmental factors seem to affect the induction of the disease [70]. Ethnicity, genetics, socio-economic factors, and eating behavior seem to increase the burden of diabetes [71]. WHO reported that people in Asia presented a lower prevalence of disease when compared with European recipients [72].

Chronic and low-grade inflammation is the angular stone of metabolic diseases, along with the lipotoxicity-mediated production of cytokines, phenotype changes of B and T cells, and macrophage infiltration into adipose tissue [71,73].

Following intestinal imbalance, decreased levels of SCFAs and the release of cytokines IL-2, IL-8, and TNF-a that promote gut inflammation are observed. Yet, the anti-inflammatory cytokine IL-10 is capable of suspending the production of pro-inflammatory cytokines, such as IFN-γ, IL-2, IL-3, and TNFα released by macrophages and Th1 cells [74]. In this regard, low amounts of IL-10 have been found in T2DM and metabolic syndrome patients [74]. The interconnection between low or altered levels of SCFAs and the prevalence of T2DM is confirmed by deviations in the lipid and glucose metabolism, which are adjusted by the GPR41 and GPR43 receptors [75]. Among SCFAs, butyric acid plays a pivotal role. Low amounts of butyric acid-producing bacteria refer to decreased glucagon and insulin levels, and increased blood glucose levels [73]. The pancreas produces more insulin to transport blood sugar into the cells, although over time, the cells stop responding to insulin and they become insulin resistant. Gut bacterial translocation to the blood may play an important role in the development of insulin resistance in T2DM [70].

T2DM is usually treated with insulin sensitizers such as thiazolidinediones (TZDs) or insulin [76], although their adverse effects limit their long use, and the research on antidiabetic hypoglycaemic medicines is consistent. Hypoglycaemic properties and the optimization of markers related to the disease have been associated with medicinal plants and herbs [77]. In this vein, a growing interest in research efforts to understand the therapeutic impact and the potential antidiabetic activities of medicinal aromatic plants [18,21,38,39,70,78,79] by the isolation of drastic compounds, biological testing of plant extracts, pharmacodynamics, toxicity testing, and finally, clinical studies, is witnessed.

Cinnamon administered as a supplement (cinnamon powder capsules) seems to significantly reduce insulin resistance in T2DM [78]. In a huge study coming from India, 156 medicinal plants and herbs showed hypoglycaemic action; while many of these herbal species contain phenols, flavonoids, phytosterols, and saponins, the ranking of the efficacy potential of those species cannot be issued due to the variety in experimental conception and methodology [77]. Yet, oxidative stress has a prominent role in diabetes mellitus (DM) [77]. Beyond their hypoglycaemic effect, many of these Indian herbal species, such as *Curcuma longa*, *Phyllanthus emblica*, and *Tinospora cordifolia*, have antioxidative properties and an impact on the regulation of DM [77].

The prevention of T2DM was induced with a high-fat diet in the C57BL/6J mouse using two hydro-alcoholic extracts of *A. herba alba Asso* (AHA) and *Centaurium erythraea Rafn* (CE), which are used in the traditional treatment of diabetes in Algeria [80], as plant extracts considerably reduce insulin resistance.

*Coreopsis tinctoria* enhanced insulin sensitivity and modulated the hepatic metabolism in high-fat diet (HFD)-induced insulin-resistant animals. Recently, the role of marein, which is a major component of *Coreopsis tinctoria*, was investigated in the improvement of insulin resistance [81].

The Mexican plant extract of *Ageratina petiolaris*, *Bromelia karatas*, *Equisetum myriochaetum*, *Rhizophora mangle*, and *Smilax moranensis* repressed the hepatic glucose production in vitro and in streptozotocin-induced diabetic rats [82].

The combined use of medicinal plants has also been studied. *Astragalus* polysaccharide (AP) and berberine (BBR) decreased insulin resistance in IR-HepG2cells, inducing major shifts in the protein burden expression (p-FoxO1Ser256 and PEPCK increase and FoxO1 and GLUT2 decrease) via the regulation of the gluconeogenesis signaling pathway [83].

A preclinical systematic review and meta-analysis study explored the protective effect of *Astragaloside* IV (AS-IV) compounds isolated from *Astragalus membranaceus* in 24 animal models of diabetic nephropathy [84]. AS-IV showed antioxidant, antiapoptotic, and antifibrotic properties.

In contrast, the ethanol extract of *Zingiber zerumbet* (EEZZ) resulted in a blood glucose decrease and an enhancement of insulin resistance in both normoglycemic and streptozotocin-induced hyperglycemic rats [85].

On the other hand, modulating the intestinal microbiota through the administration of phytomedicines led to improvements in glucose metabolism and insulin resistance in the diabetic host [86].

Nevertheless, diabetes research addresses an array of questions to obtain a profound understanding of the involved communication between intestinal microbiota and T2DM. Animal models are precious tools [70] to study these composite interactions on the pathophysiology of the disease and enable us to develop individualized phytotherapies [87] based on the modulation of the intestinal microbiota in T2DM. However, in order to extract knowledge of the mechanisms of action and efficacy and proceed to therapeutical approaches, these studies must be extrapolated, by the use of secure clinical protocols, to extended human population samples from different ethnicities and backgrounds.

Obesity is becoming an epidemic and social problem with serious health risks. WHO reports the worldwide prevalence of obesity tripling from 1975 to 2016 [87]. In 2016, more than 1.9 billion adults were overweight and over 650 million of these were obese [88]. Moreover, obesity has increased in children and adolescents to over 18% [84].

Obese people have an excess of adipose mass, producing a broad diversity of inflammatory adipocytokines, such as leptin, adiponectin, and tumor necrosis factor alpha (TNF-alpha). Inflammation, insulin resistance, and metabolic disorders coexist in obesity [87].

Medicinal plants, alone or in combination, had beneficial effects for the treatment of obesity, either through direct or indirect action. *Fucus vesiculosus*, *Citrus aurantium*, *Paullinia sorbilis*, and *Camellia thea* are acting by stimulating metabolism based on their active compounds [89]. Iodine is an active compound, issued from *Fucus vesiculosus*, with important effects in the treatment of obesity. However, its action is limited due to its untraceable impact on the thyroid gland [89]. The amine Synefrine is the active component of *Citrus Aurantium* that has an impact on the α- and β-receptors by enhancing thermogenesis, and thus, permitting a weight decrease [88,89]. Caffeine is an active component found in *Camellia thea* and *Paullinia sorbilis.* Significant effects for a weight decrease at high caffeine doses are observed, although the use of high caffeine can have deleterious effects [90].

Apart from their direct effect on metabolism, other medicinal plants featuring dietary fibers act by regulating the secretion of proopiomelanocortin (POMC) and neuropeptide Y (NPY) neurotransmitters in the hypothalamus, and thus, suppressing appetite. In this vein, *Plantago ovata* improves digestion and provides the feeling of satiety [87]. Hydroxycitric acid, a compound found in *Garcinia cambogia*, inhibits enzymes involved in the conversion of glucides to fatty masses, while gymnemic acid, found in *Gymnema sylvestre*, limits the intestinal absorption of glucose [91].

A global study coming from Brazil reported 76 plant species characterized by means of taxonomy and botany features, geographical location, chemical and biological properties for the treatment of obesity [91], and having anti-obesity action. Most plants in the families *Asteraceae* and *Fabaceae* have anti-obesity activities [91]. In this line, researchers reported that *Nigella Sativa*, *Camellia Synensis*, green tea, and black Chinese tea are linked to significant anti-obesity effects [92].

In the past, phytomedicines with diuretic activity were considered to limit water retention, although actual knowledge states that obese people without other underlying pathologies have a body water content close to that of normal individuals. Thus, diuretic phytomedicines were abandoned, considering their side effects [91].

Likewise, medicinal herbal preparations can successfully control appetite and impact feeding habits by regulating NPY and POMC neurotransmitters, and hormones such as melanocortins, a-MSH, leptin, and insulin. Yet, phytomedicines combined with a diet ensure an effective weight control.

Studies on the application of natural active compounds against different disease states are summarized in Table 2. Chronic inflammatory diseases, diabetes, allergic rhinitis, oxidative stress, and coronaviruses appear to respond in different degrees, as the pharmacological effect of each medicinal herb is the result of its different metabolites combination, as well as their synergistic effects.

## 5. Conclusions

Many natural compounds in plants have fascinating bio/pharmacological actions, as evidenced by the above research. Specifically, they possess antimicrobial, antiviral, and anti-inflammatory properties, constituting a promising source for the development of new drugs tailored to targeted therapies to combat antibiotic-resistant bacteria that cause infectious diseases such as influenza, HIV, and COVID-19, as well as chronic diseases such as diabetes, cardiovascular disorders, and cancer. However, preclinical and clinical investigations are still required in order to provide more data on their mechanisms of action, safety, and efficacy, and to better understand their therapeutic attributes, generate stronger evidence of promise for further development, and validate their effective use in medical treatments by clinical trials. Antimicrobial resistance, chronic non-communicable and infectious diseases pose a continuously growing threat for world public health, an issue that might be addressed by the extended application of medicinal plant derivatives, especially in medium- and low-income countries around the globe.

## Figures and Tables

**Table 1:** Representative studies for antimicrobial activity of plant extracts.

Plant Species/Derivative	Microbial Species	Mode of Action	Reference
*Citrus medica L. var. sarcodactylis* EO	*Escherichia coli*, *Staphylococcus aureus*, *Bacillus subtilis*, *Micrococcus luteus*	bacteria morphology, permeability of cell, and membrane integrity	[40]
*Oreganum vulgare* EO	26 *E.coli* strains and 24 *S. aureus* strains	cell wall and cytoplamic membrane disruption	[41]
*Thymus vulgaris* EO	*B. subtilis*, *E. faecalis*, and *S. aureus*. *Pseudomonas aeruginosa*, *Yersinia enterocolitica*, *Salmonella enterica* subsp. *enterica* ser. Enteritidis, *Serratia marcescens*	changes in protein profile	[42]
Cumin EO	*Escherichia coli*, *Listeria innocua*	increase in the permeabilization of the cells and disruption of the membrane integrity	[43]
*Cinnamomum zeylanycum* EO	*Escherichia coli*, *Staphylococcus aureus*	release of intracellular contents with increased levels of nucleic acids and extracellular proteins	[44]
*Citrus chagshan—huyou* EO	*Listeria monocytogenes*	morphological changes, such as wrinkled and collapsing surfaces, cavities, and fragmented cells	[45]
*Artemisia argyi* EO	*Staphylococcus aureus*	increased permeability of the cytoplasmic membrane and the extravasation of soluble proteins and intracellular potassium ions	[46]
*Chenopodium ambrosioides* L.EO and a-terpinene	*Staphylococcus aureus*	efflux pump inhibition	[47]
*Mentha piperita* EO	*Campylobacter jejuni*	elevation in the expression of general stress genes such as *dnaK*, *groEL*, and *groES*	[48]
Clove oil	*Listeria monocytogenes*	leakage of three biological macromolecules (protein, ATP, and DNA) and the reduction in two intracellular enzymes (β-galactosidase and AKP) activities	[49]
*Ocimum basilicum* L. EO	*Listeria monocytogenes*	increased cell membrane permeability, thereby causing the leakage of intracellular proteins and DNA	[50]
*Origanum vulgare* L. EO and *Leptospermum scoparium* J. R. et G. Forst EO	14 *Staphylococcus aureus* strains	collapse of the protonmotive force and depletion of the ATP pool	[51]
*Thymus* spp. EO and *Juniperus* spp. EO	*Pseudomonas hibiscicola*, *Brevibacillus agri*, *Acinetobacter calcoaceticus*	damage to the outer membrane or metabolic activities	[52]

**Table 2:** Anti-inflammatory applications of herbal compounds.

Plant Species	Active Compound	Disease	Reference
*Glycyrrhiza glabra* *Scuttelaria baicalensis*	glycyrrhizin baicalin	coronaviruses	[35]
*Camellia sinensis* (L.) Kuntze	epigallocatechin-3-gallate (EGCG) theaflavins	SARS-CoV-2	[93]
*Fabaceae* species	catechins	chronic diseases, inflammatory bowel disease (IBD),	[94]
*Horminum pyrenaicum*	diterpene quinones	suppress central Th1-type immunometabolic pathways	[95]
*Curcuma longa*	curcumin	oxidative stress	[96]
*Atropa belladona*	alcaloid atropine	anti-inflammatory, analgesic and neuro-pharmacological activities	[97]
*Centaurium erythraea* *Hibiscus rosa sinensis* *Panax ginseng*	secoiridoids, polyphenols myricetin, syringic acid ginsenosides	regulation of diabetes mellitus	[98]
*Coreopsis tinctoria*	marein	improvement of insulin resistance	[81]
*Astragalus membranaceus*	*Astragaloside* IV (AS-IV)	improvement of chemosensitivity of chemotherapy drugs	[99]
*Boswellia serrata*	3-acetyl-11-keto-β-boswellic acid (AKBBA), β-boswellic acid (BBA).	osteoarthritis	[100]
*Nigella sativa*	thymoquinone	allergic rhinitis, metabolic disorders, diabetes mellitus	[101]
*Spatholobus suberectus*, *Dioscorea nipponica*, and *Zingiber officinale*	biochanin A and 6-gingerol	pro-inflammatory cytokines and MAPKs signaling pathway	[102]
*Crateva adansonii*, *Maerua siamensis*, and *Mallotus repandus*	lupeol	analgesic	[103]

## Data Availability

Not applicable.
